# Developing risk prediction models for type 2 diabetes: a systematic review of methodology and reporting

**DOI:** 10.1186/1741-7015-9-103

**Published:** 2011-09-08

**Authors:** Gary S Collins, Susan Mallett, Omar Omar, Ly-Mee Yu

**Affiliations:** 1 Centre for Statistics in Medicine, University of Oxford, Wolfson College Annexe, Linton Road, Oxford, OX2 6UD, UK

## Abstract

**Background:**

The World Health Organisation estimates that by 2030 there will be approximately 350 million people with type 2 diabetes. Associated with renal complications, heart disease, stroke and peripheral vascular disease, early identification of patients with undiagnosed type 2 diabetes or those at an increased risk of developing type 2 diabetes is an important challenge. We sought to systematically review and critically assess the conduct and reporting of methods used to develop risk prediction models for predicting the risk of having undiagnosed (prevalent) or future risk of developing (incident) type 2 diabetes in adults.

**Methods:**

We conducted a systematic search of PubMed and EMBASE databases to identify studies published before May 2011 that describe the development of models combining two or more variables to predict the risk of prevalent or incident type 2 diabetes. We extracted key information that describes aspects of developing a prediction model including study design, sample size and number of events, outcome definition, risk predictor selection and coding, missing data, model-building strategies and aspects of performance.

**Results:**

Thirty-nine studies comprising 43 risk prediction models were included. Seventeen studies (44%) reported the development of models to predict incident type 2 diabetes, whilst 15 studies (38%) described the derivation of models to predict prevalent type 2 diabetes. In nine studies (23%), the number of events per variable was less than ten, whilst in fourteen studies there was insufficient information reported for this measure to be calculated. The number of candidate risk predictors ranged from four to sixty-four, and in seven studies it was unclear how many risk predictors were considered. A method, not recommended to select risk predictors for inclusion in the multivariate model, using statistical significance from univariate screening was carried out in eight studies (21%), whilst the selection procedure was unclear in ten studies (26%). Twenty-one risk prediction models (49%) were developed by categorising all continuous risk predictors. The treatment and handling of missing data were not reported in 16 studies (41%).

**Conclusions:**

We found widespread use of poor methods that could jeopardise model development, including univariate pre-screening of variables, categorisation of continuous risk predictors and poor handling of missing data. The use of poor methods affects the reliability of the prediction model and ultimately compromises the accuracy of the probability estimates of having undiagnosed type 2 diabetes or the predicted risk of developing type 2 diabetes. In addition, many studies were characterised by a generally poor level of reporting, with many key details to objectively judge the usefulness of the models often omitted.

## Background

The global incidence of type 2 diabetes is increasing rapidly. The World Health Organisation predicts that the number of people with type 2 diabetes will double to at least 350 million worldwide by 2030 unless appropriate action is taken [1]. Diabetes is often associated with renal complications, heart disease, stroke and peripheral vascular disease, which lead to increased morbidity and premature mortality, and individuals with diabetes have mortality rates nearly twice as high as those without diabetes [2]. Thus the growing healthcare burden will present an overwhelming challenge in terms of health service resources around the world. Early identification of patients with undiagnosed type 2 diabetes or those at an increased risk of developing type 2 diabetes is thus a crucial issue to be resolved.

Risk prediction models have considerable potential to contribute to the decision-making process regarding the clinical management of a patient. Typically, they are multivariable, combining several patient risk predictors that are used to predict an individual's treatment outcome. Healthcare interventions or lifestyle changes can then be targeted towards those at an increased risk of developing a disease. Similarly, the function of these models can also be to screen individuals to identify those who are at an increased risk of having an undiagnosed condition, for which diagnosis management and treatment can be initiated and ultimately improve patient outcomes.

However, despite the large number of risk prediction models being developed, only a very small minority end up being routinely used in clinical practice. Reasons for the uptake of one risk prediction model and not another is unclear, though poor design, conduct and ultimately reporting will inevitably be leading causes for apprehension. Lack of objective and unbiased evaluation (validation) is a clear concern, but also, when performance is evaluated, poor performance data to support the uptake of a risk prediction model can contribute to scepticism regarding the reliability and ultimately the clinical usefulness of a model. Dictating the performance is how the risk prediction model was originally developed.

There is a growing concern that the majority of risk prediction models are poorly developed because they are based on a small and inappropriate selection of the cohort, questionable handling of continuous risk predictors, inappropriate treatment of missing data, use of flawed or unsuitable statistical methods and, ultimately, a lack of transparent reporting of the steps taken to derive the model [3-12].

Whilst a number of guidelines in the medical literature exist for the reporting of randomised, controlled trials [13], observational studies [14], diagnostic accuracy [15], systematic reviews and meta-analyses [16] and tumour marker prognostic studies [17], there are currently no consensus guidelines for developing and evaluating multivariable risk prediction models in terms of conduct or reporting. Although a number of texts and guidance exist that cover many of the issues in developing a risk prediction model [18-20], these are spread across the literature at varying levels of prior knowledge and expertise. Raising the quality of studies is likely to require a single, concise resource for easy use by authors, peer reviewers and ultimately consumers of risk prediction models to objectively evaluate the reliability and usefulness of new risk prediction models. Furthermore, there is currently no guidance on what aspects of model development and validation should be reported so that readers can objectively judge the value of the prediction model.

The aim of this article is to review the methodological conduct and reporting of articles deriving risk prediction models for predicting the risk of having undiagnosed (prevalent) type 2 diabetes or the future risk of developing (incident) type 2 diabetes.

## Methods

We identified articles that presented new risk prediction models for predicting the risk of detecting undiagnosed (prevalent) diabetes or predicting the risk of developing (incident) type 2 diabetes. The PubMed and EMBASE databases were initially searched on 25 February 2010 (a final search was conducted on 13 May 2011). The search string is given in Appendix 1. Articles were restricted to the English-language literature. Searches included articles from all years in the PubMed (from 1965) and EMBASE (from 1980) databases. Additional articles were identified by searching the references in papers identified by the search strategy and our own personal reference lists.

### Inclusion criteria

Articles were included if they met our inclusion criteria: the primary aim of the article had to be the development of a multivariable (more than two variables) risk prediction model for type 2 diabetes (prediabetes, undiagnosed diabetes or incident diabetes). Articles were excluded if (1) they included only validation of a preexisting risk prediction model (that is, the article did not develop a model), (2) the outcome was gestational diabetes, (3) the outcome was type 1 diabetes, (4) participants were children or (5) the authors developed a genetic risk prediction model.

### Data extraction, analysis and reporting

One person (GSC) screened the titles and abstracts of all articles identified by the search string to exclude articles not pertaining to risk prediction models. Items were recorded by duplicate data extraction by combinations of two from four reviewers (GSC, SM, LMY and OO). One reviewer (GSC) assessed all articles and all items, whilst the other reviewers collectively assessed all articles (SM, LMY and OO). Articles were assigned to reviewers (SM, LMY and OO) in a random manner using variable block randomisation. In articles that presented more than one model, the model that was recommended by the authors was selected. No study protocol is available. Data items extracted for this review include study design, sample size and number of events, outcome definition, risk predictor selection and coding, missing data, model-building strategies and aspects of performance. The data extraction form for this article was based largely on two previous reviews of prognostic models in cancer [3,21,22] and can be obtained on request from the first author (GSC).

For the primary analysis, we calculated the proportion of studies and, where appropriate, the number of risk prediction models for each of the items extracted. We have reported our systematic review in accordance with the PRISMA guidelines [16], with the exception of items relating to meta-analysis, as our study includes no formal meta-analysis.

## Results

**Figure 1:**
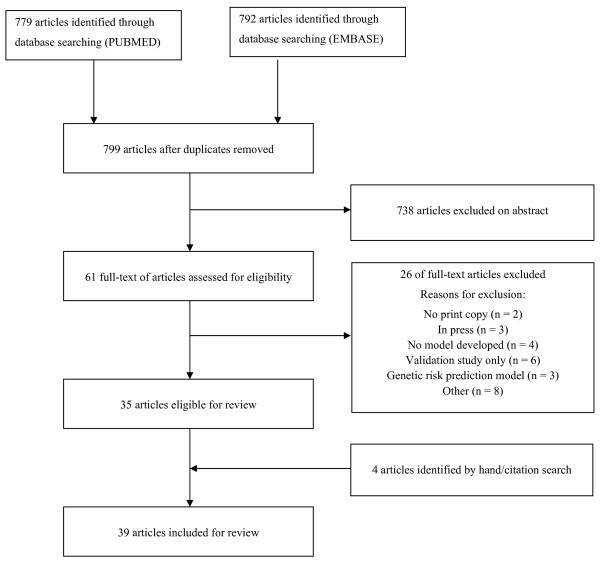
**Flow diagram of selected studies**.

The search string retrieved 779 articles in PubMed and 792 articles in EMBASE, and, after removing duplicates, our database search yielded 799 articles (see Figure 1). Thirty-five articles met our inclusion criteria, and a further four articles were retrieved by hand-searching reference lists or citation searches. In total, 39 studies were eligible for review, among which 32 studies (83%) were published between January 2005 and May 2011. Thirteen studies (33%) were published in *Diabetes Care*, five studies (13%) were published in *Diabetes Research and Clinical Practice*, four studies (10%) were published in *Diabetic Medicine *and three studies (8%) were published in the *Annals of Internal Medicine*. Four studies reported separate risk prediction models for men and women [23-26], thus our review assesses a total of 43 risk prediction models from 39 articles. Thus the denominator is 39 when reference is made to studies and 43 when reference is made to risk prediction models. The outcomes predicted by the models varied because of different definitions of diabetes and patients included (Tables 1, 2 and 3). Seventeen studies (44%) described a model to predict the development of diabetes (incident diabetes) [23,25,27-40], fifteen (38%) described the development of a model to predict the risk of having undiagnosed diabetes [41-53], four described the development of a prediction model for diagnosed and undiagnosed diabetes [24,26,54,55], one described the development of a prediction model for undiagnosed diabetes and prediabetes [56], one described the development of a prediction model for abnormal postchallenge plasma glucose level (defined as ≥ 140 mg/dL) to predict undiagnosed diabetes [57] and one described the development of a model to predict the risk of undiagnosed type 2 diabetes and impaired glucose regulation [58].

**Table 1:** Models for predicting risk of incident diabetes^a^

Study	Year	Country	Definition of diabetes as reported	Risk predictors in the model
Aekplakorn *et al. *[27]	2006	Thailand	Diabetes diagnosed according to ADA criteria as FPG level ≥ 126 mg/dL (7.0 mmol/L) or 2-h PG level ≥ 200 mg/dL (11.1 mmol/L) or a previous diagnosis of diabetes	Age, sex, BMI, abdominal obesity (waist circumference), hypertension, family history of diabetes
Balkau *et al. *[23]	2008	France	Incident cases of diabetes identified by treatment for diabetes or FPG ≥ 7.0 mmol/L	Men: waist circumference, smoking status, hypertension. Women: waist circumference, family history of diabetes, hypertension.
Chen *et al. *[28]	2010	Australia	Incident diabetes at follow-up defined by treatment with insulin or oral hypoglycaemic agents, FPG level ≥ 7.0 mmol/L, or 2-hPG in OGTT ≥ 11.1 mmol/L	Age, sex, ethnicity, parental history of diabetes, history of high blood glucose, use of antihypertensive medication, smoking status, physical activity, waist circumference
Chien *et al. *[29]	2009	Taiwan	Diabetes defined by FPG ≥ 7.0 mmol/L or use of oral hypoglycaemic or insulin medication	Age, BMI, WBC count, and triacylglycerol, HDL cholesterol, FPG levels
Gao *et al. *[30]	2009	Mauritius	Diabetes diagnosed according to 2006 WHO/IDF criteria. Diabetes cases were defined as those who reported a history of diabetes and treatment with glucose-lowering medication and/or FPG ≥ 7.0 mmol/L and/or 2-h PG ≥ 11.1 mmol/L.	Age, sex, BMI, waist circumference, family history of diabetes
Gupta *et al. *[40]	2008	UK, Ireland, Sweden, Denmark, Iceland, Norway, Finland	FPG ≥ 7 mmol/L or random glucose ≥ 11.1 mmol/L at randomisation or screening visits. Self-reported history of diabetes and drug or dietary therapy for diabetes. Presence of both impaired FPG (> 6 and < 7 mmol/L) and glycosuria at randomisation or screening visits.	Age, sex, FPG, BMI, randomised group, triglycerides, systolic blood pressure, total cholesterol, use of non-coronary artery disease medication, HDL cholesterol, alcohol intake
Hippisley-Cox *et al. *[25]	2009	UK	Patients with diabetes identified by searching electronic health records for diagnosis Read code for diabetes (C10%)	Age, BMI, family history of diabetes, smoking status, treated hypertension, current treatment with corticosteroids, diagnosis of CVD, social deprivation, ethnicity
Kahn *et al. *[31]	2009	USA	Participants were considered to have diabetes if they reported a history of physician-diagnosed 'diabetes (sugar in the blood)' or if their FPG level was ≥ 7.0 mmol/L (≥ 126 mg/dL), their non-FPG level was at least 11.1 mmol/L (≥ 200 mg/dL), or their 2-h PG at year 9 follow-up was ≥ 11.1 mmol/L (≥ 200 mg/dL). Additional cases of incident diabetes were identified by criteria-based abstractions of hospital records.	Diabetic mother, diabetic father, hypertension, ethnicity, age, smoking status, waist circumference (sex), height (sex), resting pulse (sex), weight (sex)
Kolberg *et al. *[32]	2009	Denmark	Diagnosis of type 2 diabetes was defined by 2-h PG ≥ 11.1 mmol/L on OGTT or FPG ≥ 7.0 mmol/L	Adiponectin, C-reactive protein, ferritin, interleukin 2 receptor A, glucose, insulin
Lindström *et al. *[33]	2003	Finland	Subjects not on antidiabetic drug treatment were diagnosed as having diabetes according to WHO 1999 criteria [12] if they had FPG ≥ 7.0 mmol/L (fasting whole blood glucose ≥ 6.1 mmol/L) and/or 2-h PG ≥ 11.1 mmol/L (2-h whole blood glucose ≥ 10.0 mmol/L)	Age, BMI, waist circumference, use of blood pressure medication, history of high blood glucose, physical activity, daily consumption of vegetables
Liu *et al. *[61]	2011	China	Diabetes was diagnosed according to ADA criteria as FPG ≥ 126 mg/dL (7.0 mmol/L) or OGTT ≥ 200 mg/dL (11.1 mmol/L). Incident diabetes was ascertained from multiple sources: self-report, FPG and OGTT results, and data on prescribing of hypoglycaemic medication at follow-up survey.	Age, hypertension, history of high blood glucose, BMI, high FPG
Schmidt *et al. *[34]	2005	USA	Incident diabetes defined by OGTT (FPG ≥ 7.0 mmol/L or a 2-h PG ≥ 11.1 mmol/L) at end of follow-up (1996 to 1998) or as report of clinical diagnosis or treatment for diabetes during follow-up period	Age, ethnicity, parental history of diabetes, FPG, systolic blood pressure, waist circumference, height, HDL cholesterol, triglycerides
Schulze *et al. *[35]	2007	Germany	Incident diabetes identified through August 2005 by self-reports of diabetes diagnosis, diabetes relevant medication or dietary treatment due to diabetes. All cases were verified by diagnosing physician on basis of ICD-10 criteria.	Waist circumference, height, age, hypertension, intake of red meat, intake of whole-grain bread, coffee consumption, alcohol consumption, physical activity, former smoker, current heavy smoker (≥ 20 cigarettes/day
Stern *et al. *[36]	2002	USA	Diabetes diagnosed according to WHO criteria (FPG ≥ 7.0 mmol/L (≥ 126 mg/dL) or 2-h PG ≥ 11.1 mmol/L (≥ 200 mg/dL)) [3]. Persons who reported history of diabetes diagnosed by physician and reported current use of insulin or oral antidiabetic agent were considered to have diabetes regardless of plasma glucose level.	Age, sex, ethnicity, FPG, systolic blood pressure, HDL cholesterol, BMI, family history of diabetes
Sun *et al. *[37]	2009	Taiwan	Not defined	Sex, education level, age, current smoking status, BMI, waist circumference, family history of diabetes, hypertension, FPG
Tuomilehto *et al. *[38]	2010	Canada, Germany, Austria, Norway, Denmark, Sweden, Finland, Israel, Spain	Primary end point was development of type 2 diabetes, defined as a 2-h PG ≥ 11.1 mmol/L	Acarbose treatment, sex, serum triglyceride level, waist circumference, FPG, height, history of CVD, diagnosed hypertension
Wilson *et al. *[39]	2007	USA	Participants characterised as developing new diabetes during follow-up if they (1) started receiving oral hypoglycaemic agents or insulin or (2) had a FPG ≥ 126 mg/dL (≥ 7.0 mmol/L)	FPG, BMI, HDL cholesterol, parental history of diabetes, triglyceride level, blood pressure

^a^ADA, American Diabetes Association; BMI, body mass index; WBC, white blood cell; HDL, high-density lipoprotein; WHO/IDF, World Health Organisation/International Diabetes Federation; FPG, fasting plasma glucose; OGTT, oral glucose tolerance test; ICD-10, International Statistical Classification of Diseases and Related Health Problems 10th Revision; CVD, cardiovascular disease; 2-h PG, two-hour 75-g postload plasma glucose level.

**Table 2:** Models for predicting risk of prevalent (undiagnosed) diabetes^a^

Study	Year	Country	Definition of diabetes as reported	Risk predictors in the model
Al Khalaf *et al. *[60]	2010	Kuwait	Diagnosis of diabetes based on ADA 2003 criteria. If FPG was ≥ 7.0 mmol/L or random glucose was ≥ 11.1 mmol/L, participants were classified as having newly diagnosed diabetes.	Age, waist circumference, blood pressure medication, diabetes in sibling
Al-Lawati *et al. *[41]	2007	Oman	Diabetes was diagnosed according to 1998 WHO criteria for OGTT (FPG 11.1 mmol/l 2-h post 75-g glucose load	Age, waist circumference, BMI, family history of diabetes, hypertension
Baan *et al. *[42]	1999	The Netherlands	Diabetes defined as use of antidiabetic medication (insulin or oral hypoglycaemic medication) and/or 2-h PG ≥ 11.1 mmol/L according to WHO criteria	Age, sex, use of antihypertensive medication, obesity (BMI ≥ 30)
Bang *et al. *[43]	2009	USA	Undiagnosed diabetes defined as FPG ≥ 7.0 mmol/L (≥ 126 mg/dL)	Age, sex, family history of diabetes, history of hypertension, obesity (BMI or waist circumference), physical activity
Borrell *et al. *[59]	2007	USA	FPG ≥ 126 mg/dL	Age, sex, ethnicity, family history of diabetes, self-reported hypertension, hypercholesterolaemia, periodontal disease
Chaturvedi *et al. *[44]	2008	India	Undiagnosed diabetes defined as those with FPG ≥ 126 mg/dL (≥ 7.0 mmol/L) but who were not aware of their glycaemic status	Age, blood pressure, waist circumference, family history of diabetes
Gao *et al. *[45]	2010	China	Diabetes defined according to 2006 WHO/IDF criteria. In individuals without known diabetes, undiagnosed diabetes was determined if person had FPG ≥ 7.0 mmol/L and/or postchallenge PG ≥ 11.1 mmol/L	Age, waist circumference, family history of diabetes
Glümer *et al. *[46]	2004	Denmark	Individuals without known diabetes and with FPG ≥ 7.0 mmol/L or 2-h PG ≥ 11.1 mmol/L defined as having SDM	Age, BMI, sex, known hypertension, physical activity, family history of diabetes
Keesukphan *et al. *[47]	2007	Thailand	75-g OGTT carried out as outlined by WHO Diabetes Study Group	Age, BMI, history of hypertension
Ko *et al. *[48]	2010	Hong Kong	All subjects underwent 75-g OGTT using 1998 WHO criteria (FPG ≥ 7.0 mmol/L and/or 2-h PG ≥ 11.1 mmol/L	Age, sex, BMI, hypertension, dyslipidaemia, family history of diabetes, gestational diabetes
Mohan *et al. *[49]	2005	India	Diagnosis of diabetes based on WHO Consulting Group criteria, that is, 2-hr PG ≥ 200 mg/dL	Age, abdominal obesity (waist circumference), physical activity, family history of diabetes
Pires de Sousa *et al. *[50]	2009	Brazil	FPG > 126 mg/dL (7.0 mmol/L), that is, provisional diagnosis of diabetes according to ADA criteria, classified as type 2 diabetes patients	Age, BMI, hypertension
Ramachandran *et al. *[51]	2005	India	Diabetes diagnosis based on 2-h PG ≥ 11.1 mmol/L	Age, family history of diabetes, BMI, waist circumference, physical activity
Ruige *et al. *[52]	1997	The Netherlands	Participants underwent 75-g OGTT and were classified according to WHO criteria	Frequent thirst, pain during walking with need to slow down, shortness of breath when walking, age, sex, obesity (BMI), obesity (men), family history of diabetes, use of antihypertensive drugs, reluctance to use bicycle for transportation
Tabaei and Herman [53]	2002	Egypt	Undiagnosed diabetes defined based on FPG ≥ 126 mg/dL and/or 2-h PG ≥ 200 mg/dL	Age, random plasma glucose, postprandial time, sex, BMI

^a^SDM, screen-detected diabetes; ADA, American Diabetes Association; BMI, body mass index; WHO/IDF, World Health Organisation/International Diabetes Federation; FPG, fasting plasma glucose; OGTT, oral glucose tolerance test; 2-h PG, two-hour 75-g postload plasma glucose level.

**Table 3:** Models for predicting risk of other diabetes outcomes^a^

Study	Year	Country	Model objective (undiagnosed or incident diabetes)	Definition of diabetes as reported	Risk predictors in the model
Bindraban *et al. *[54]	2008	The Netherlands	Diagnosed and undiagnosed	FPG ≥ 7.0 mmol/L and/or self-report	Age, BMI, waist circumference, resting heart rate, first-degree relative with diabetes, hypertension, history of CVD, ethnicity
Cabrera de León *et al. *[24]	2008	Canary Islands	Unclear	Persons recorded as having diabetes if they said they had the disease and reported dietary or pharmacological treatment with oral antidiabetics or insulin. Persons were considered to have undetected type 2 diabetes if they were unaware of disease at time of inclusion in study but had two consecutive FPG values ≥ 7 mmol/L (≥ 126 mg/dL).	Men: age, waist/height ratio, family history of diabetes Women: age, waist/height ratio, family history of diabetes, gestational diabetes
Gray *et al. *[58]	2010	UK	Undiagnosed and impaired glucose regulation	Participants diagnosed with type 2 diabetes according to WHO criteria [1] with FPG ≥ 7 mmol/L and/or 2-h PG ≥ 11.1 mmol/L. IFG defined as FPG between 6.1 and 6.9 mmol/L inclusive.	Age, ethnicity, sex, first-degree family history of diabetes, antihypertensive therapy or history of hypertension, waist circumference, BMI
Griffin *et al. *[55]	2000	UK	Diagnosed and undiagnosed	Classified according to WHO criteria	Sex, prescribed antihypertensive medication, prescribed steroids, age, BMI, family history of diabetes, smoking status
Heikes *et al. *[56]	2008	USA	Undiagnosed and pre-diabetes	Diabetes is defined as FPG ≥ 126 mg/dL and/or 2-h OGTT ≥ 200 mg/dL. Prediabetes defined as IFG and/or IGT without diabetes. Undiagnosed diabetes defined as presence of actual diabetes based on FPG and/or 2-h OGTTand absence of having been told that he or she has diabetes.	Age, waist circumference, history of gestational diabetes, family history of diabetes, ethnicity, high blood pressure, weight, height, parental diabetes, exercise
Kanaya *et al. *[57]	2005	USA	Abnormal PCPG	Abnormal 2-h PG postchallenge test result (≥ 140 mg/dL)	Sex, age, triglycerides, FPG
Xie *et al. *[26]	2010	China	Diagnosed and undiagnosed	Participants without a previous diagnosis of diabetes were categorised according to the ADA diagnostic criteria as follows: undiagnosed diabetes (FPG ≥ 7.0 mmol/L) and impaired fasting glycaemia (6.1 to 6.9 mmol/L). Diabetes was defined as self-reported history of diabetes plus undiagnosed diabetes.	Men: waist circumference, age Women: waist/hip ratio, age

^a^ADA, American Diabetes Association; BMI, body mass index; WHO, World Health Organisation; FPG, fasting plasma glucose; OGTT, oral glucose tolerance test; CVD, cardiovascular disease; 2-h PG, two-hour 75-g postload plasma glucose level; IGT, impaired glucose tolerance; IFG, impaired fasting glucose.

In terms of geography, all but two risk prediction models were developed using patient data from single countries [38,40]. Eight articles (21%) were from the USA [31,34,36,39,43,56,57,59], thirteen articles (33%) were from Europe [23-25,32,33,35,40,42,46,52,54,55], thirteen articles (33%) were from Asia [26,27,29,37,41,44,45,47-49,51,60], two were from Africa [30,53], one was from Australia [28] and one was from Brazil [50].

### Number of patients and events

The number of participants included in developing risk prediction models was clearly reported in 35 (90%) studies. In the four studies where this was not clearly reported, the number of events was not reported [26,34,49,56]. The median number of participants included in model development was 2,562 (interquartile range (IQR) 1,426 to 4,965). One particular study that included 2.54 million general practice patients used separate models for men (1.26 million) and women (1.28 million) [25]. Six studies (15%) did not report the number of events in the analysis [26,34,47,49,56,58]. Where the number of events was recorded, the median number of events used to develop the models was 205 (IQR 135 to 420).

### Number of risk predictors

**Figure 2:**
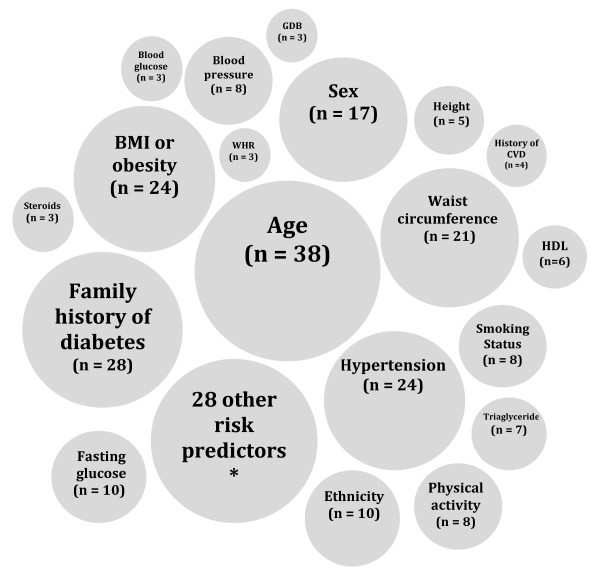
**Frequency of identified risk predictors in the final prediction models**. * Other risk predictors appearing no more than twice in the final model; (1) white blood cell. count, (2) dyslipidaemia, (3) adiponectin, (4) C-reactive protein, (5) ferritin, (6) interleuken-2 receptor A, (7) insulin, (8) glucose, (9) vegetable consumption, (10) frequent thirst, (11) pain during walking, (12) shortness of breath, (13) reluctance to use bicycle, (14) total cholesterol, (15) intake of red meat, (16) intake of whole-grain bread, (17) coffee consumption, (18) educational level, (19) postprandial time, (20) non-coronary artery disease medication, (21) acarbose treatment, (22) hypercholesterolemia, (23) periodontal disease, (24) RCT group *[1-24 all appear only once]*, (25) alcohol consumption (26) resting heart rate, (27) weight, (28) social deprivation *[25-28 appear twice] *Abbreviations: WHR = waist-to-hip ratio; HDL = High density lipoprotein; GDB = Gestational diabetes.

The number of candidate risk predictors was not reported or was unclear in seven studies [27,31,37,47,48,52,54,60]. A median of 14 risk predictors (IQR 9 to 19, range 4 to 64) were considered candidate risk predictors. The rationales or references for including risk predictors were provided in 13 studies [25,29,31,32,38,42,46,49-52,56,58]. The final reported prediction models included a median of six risk predictors (IQR 4 to 8, range 2 to 11). In total, 47 different risk predictors were included in the final risk prediction models (see Figure 2). The most commonly identified risk predictors included in the final risk prediction model were age (*n *= 38), family history of diabetes (*n *= 28), body mass index (*n *= 24), hypertension (*n *= 24), waist circumference (*n *= 21) and sex (*n *= 17). Other commonly identified risk predictors included ethnicity and fasting glucose level (both *n *= 10) and smoking status and physical activity (both *n *= 8). Twenty-four risk predictors appeared only once in the final risk prediction model.

### Sample size

The number of events per variable could not be calculated for 14 models. Nine risk prediction models (21%) were developed in which the number of events per variable was < 10. Overall, the median number of events per variable was 19 (IQR 8 to 36, range 2.5 to 4,796).

### Treatment of continuous risk predictors

Thirteen prediction models (30%) were developed retaining continuous risk predictors as continuous, twenty-one risk prediction models (49%) dichotomised or categorised all continuous risk predictors and six risk prediction models (14%) kept some continuous risk predictors as continuous and categorised others (Table 4). It was unclear how continuous risk predictors were treated in the development of three risk prediction models (7%). Only five studies (13%) considered nonlinear terms [23,25,34,35,40], of which only the QDScore Diabetes Risk Calculator included nonlinear terms in the final prediction model [25].

**Table 4:** Issues in model development^a^

Variables	Data
Sample size, median (IQR)	
Development cohort^b^	2,562 (1,426 to 4,965)
Validation cohorts^c^	1,895 (1,253 to 4,398)
	
Treatment of continuous risk predictors, *n *(%)	
All kept continuous	13 (30%)
All categorised/dichotomised	21 (49%)
Some categorised, some not	6 (14%)
Unclear	3 (7%)
	
Treatment of missing data, *n *(%)	
Not mentioned	16 (41%)
Complete case	21 (54%)
Multiple imputation	1 (3%)
Other (for example, surrogate splitter for regression trees)	1 (3%)
	
Model-building strategy, *n *(%)	
Stepwise, forward selection, backward elimination	20 (51%)
All significant in univariate analysis	2 (5%)
Other	12 (31%)
Unclear	5 (13%)
	
Overfitting mentioned or discussed, *n *(%)	5 (13%)

^a^IQR, interquartile range; ^b^sample size not reported in four studies; ^c^sample size not reported in two studies and unclear in one study.

### Missing data

Twenty-three studies (59%) made reference to missing data in developing the risk prediction model, of which twenty-one studies explicitly excluded individuals with missing data regarding one or more risk predictors (often a specified inclusion criterion), thereby rendering them complete case analyses [23,26,28-31,33-38,40,41,43-46,54,58,61]. One study derived the model using a complete case approach, though it included a sensitivity analysis to examine the impact of missing data [58]. One study used multiple imputations to replace missing values for two risk predictors [25]. One study used two different approaches to developing a risk prediction model (logistic regression and classification trees) with surrogate splitters to deal with missing data when using classification trees, whilst the approach for dealing with missing data in the logistic regression analyses was not reported, in which event a complete case analysis was most likely.. Sixteen studies (41%) made no mention of missing data (Table 4), thus it can only be assumed that a complete case analysis was conducted or that all data for all risk predictors (including candidate risk predictors) were available, which seems unlikely [24,27,32,39,42,47-53,55,57,59,60].

### Model building

Eight studies (21%) reported using bivariable screening (often referred to as 'univariate screening') to reduce the number of risk predictors [32,34,44-46,50,52,54], whilst it was unclear how the risk predictors were reduced prior to development of the multivariable model in nine studies (23%) [23,29,31,35,37,47,48,55,58]. Two studies reported examining the association of individual risk predictors with patient outcome after adjusting for age and sex [27] and age and cohort [30]. Nine studies (23%) included all risk predictors in the multivariable analysis [25,26,33,36,39,49,51,53,61].

Twenty-two studies (56%) reported using automated variable selection (forward selection, backward elimination and stepwise) procedures to derive the final multivariable model (Table 4). Nine studies (23%) reported using backward elimination [24,28,41,43,45,46,50,52,57], seven studies (18%) reported using forward selection [34,35,38,40,48,55,60] whilst six studies (15%) used stepwise selection methods [23,32,42,47,54,58].

All studies clearly identified the type of model they used to derive the prediction model. The final models were based on logistic regression in 29 articles, the Cox proportional hazards model in 7 articles [25,29,30,35,37,38,40], recursive partitioning in 2 articles [26,56] and a Weibull parametric survival model in 1 article [31]. Two studies used two modelling approaches (logistic regression and Cox proportional hazards model [39] and logistic regression and recursive partitioning [56]).

Twenty-five risk prediction models (58%) considered interactions in developing the model; however, this was not explicitly stated for seven of these risk prediction models. Three studies clearly stated that they did not consider interactions to keep the risk prediction model simple, yet all three models implicitly included a waist circumference by sex interaction in their definition of obesity [33,41,44]. Two studies examined over 20 interactions [36,43].

### Validation

Ten studies (26%) randomly split the cohort into development and validation cohorts [24-26,30,31,34,37,46,51,55] (Table 5). Eight of these studies split the original cohort equally into development and validation cohorts. Twenty-one studies (54%) conducted and published an external validation of their risk prediction models within the same article [23,27,28,33,35,38,41-48,50-53,56-58], and eight of these studies used two or more data sets in an attempt to demonstrate the external validity (that is, generalisability) of the risk prediction model.

**Table 5:** Evaluating performance of risk prediction models^a^

Parameter	Number of studies (%)
Validation	
Apparent	30 (77%)
Internal	15 (38%)
Bootstrapping	2 (5%)
Jack-knifing	1 (3%)
Random split sample	10 (26%)
Cross-validation	2 (5%)
Temporal	3 (8%)
External	21 (54%)
	
Performance metrics^b^	
Discrimination	
C-statistic	39 (100%)
D-statistic	1 (3%)
Calibration^c^	10 (26%)
Hosmer-Lemeshow statistic	8 (21%)
Calibration plot	2 (5%)
Classification	
Reclassification (NRI)	2 (5%)
Other (for example, sensitivity, specificity)	31 (79%)

^a^NRI,- Net Reclassification Index; ^b^studies can report more than one performance metric; ^c^calibration assessed on the basis of the development cohort in 10 studies and in the validation cohorts in 2 studies.

### Model performance

We assessed the type of performance measure used to evaluate the risk prediction models (Table 5). All studies reported C-statistics, with 31 studies (79%) reporting C-statistics on the data used to derive the model [23,26-29,32,33,35-39,41,43-54,56-61], 13 studies (33%) calculating C-statistics on an internal validation data set [24-26,29-32,34,37,39,40,55,56] and 21 studies (54%) reporting C-statistics on external validation data sets [23,27,28,33,35,38,41-48,50-53,56-58]. Only 10 studies (26%) assessed how well the predicted risks compared to the observed risks (calibration), investigators in 8 studies (21%) chose to calculate the Hosmer-Lemeshow goodness-of-fit test [23,27-29,36,37,45,53] and in 2 studies a calibration plot was presented [25,37].

### Model presentation

Twenty-four studies (62%) derived simplified scoring systems from the risk models [23,24,27-29,31,33,38,39,41-46,48-52,57,58,61]. Twelve studies derived a simple points system by multiplying (or dividing) the regression coefficients by a constant (typically 10) and then rounding the result to the nearest integer [24,41-44,46,48,50-52,57,58]. Four studies used the method of Sullivan *et al. *[62] to develop a points system [27,29,38,39].

## Discussion

### Main findings

Our systematic review of 39 published studies highlights inadequate conduct and reporting in all aspects of developing a multivariable prediction model for detecting prevalent or incident type 2 diabetes. Fundamental aspects of describing the data (i.e. the number of participants and the number of events), a clear description of all selection of risk predictors and steps taken to build the multivariable model were all shown to be poor

One of the problems researchers face when developing a multivariable prediction model is overfitting. This occurs when the number of events in the cohort is disproportionately small in relation to the number of candidate risk predictors. A rule of thumb is that models should be developed with 10 to 20 events per variable (EPV) [63,64]. Of the studies included in this review, 21% had fewer than 10 EPV, whilst there was insufficient detail reported for an EPV to be calculated in 33% of the risk prediction models. The consequences of overfitting are that models subsequently often fail to perform satisfactorily when applied to data sets not used to derive the model [65]. Investigators in other studies have reported similar findings (EPV < 10) when appraising the development of multivariable prediction models [3,21,66].

Another key component affecting the performance of the final model is how continuous variables are treated, whether they are kept as continuous measurements or whether they have been categorised into two or more categories [67]. Common approaches include dichotomising at the median value or choosing an optimal cutoff point based on minimising a *P *value. Regardless of the approach used, the practice of artificially treating a continuous risk predictor as categorical should be avoided [67], yet this is frequently done in the development of risk prediction models [4,5,68-74]. In our review, we identified 63% of studies that categorised all or some of the continuous risk predictors, and similar figures have been reported in other reviews [3]. Dichotomising continuous variables causes a detrimental loss of information and loss of power to detect real relationships, equivalent to losing one-third of the data or even more if the data are exponentially distributed [75]. Continuous risk predictors (that is, age) should be retained in the model as continuous variables, and if the risk predictor has a nonlinear relationship with the outcome, then the use of splines or fractional polynomial functions is recommended [76].

Missing data is common in most clinical data sets, which can be a serious problem in studies deriving a risk prediction model. Regardless of study design, collecting all data on all risk predictors for all individuals is a difficult task that is rarely achieved. For studies that derive models on the basis of retrospective cohorts, there is no scope in retrieving any missing data and investigators are thus confronted with deciding how to deal with incomplete data. A common approach is to exclude individuals with missing values on any of the variables and conduct a complete case analysis. However, a complete case analysis, in addition to sacrificing and discarding useful information, is not recommended as it has been shown that it can yield biased results [77]. Forty percent of the studies in our review failed to report any information regarding missing data. Multiple imputation offers investigators a valid approach to minimise the effect of missing data, yet this is seldom done in developing risk prediction models [78], though guidance and illustrative examples are slowly appearing [18,79,80]. The completeness of overall data (how many individuals have complete data on all variables) and by variable should always be reported so that readers can judge the representativeness and quality of the data.

Whilst developing a model, predictors that are shown to have little influence on predicting patients likely to have particular outcomes might be taken out of a final model during model development. However, this is not a simple matter of selecting predictors solely on the basis of statistical significance during model development, as it can be important to retain these among the model risk predictors known to be important from the literature, but which may not reach statistical significance in a particular data set. Unfortunately, the process of developing a risk predictor model for use in clinical practice for prediction is often confused with using multivariate modelling to identify risk predictors with statistical significance in epidemiological studies. This misunderstanding of the modelling aims can lead to use of inappropriate methods such as prescreening candidate variables for a risk predictor model based on bivariable tests of association with the outcome (that is, a statistical test to examine the association of an individual predictor with the outcome). This has been shown to be inappropriate, as it can wrongly reject important risk predictors that become prognostic only after adjustment of other risk predictors, thus leading to unreliable models [18,81]. More importantly, it is crucial to clearly report any procedure used to reduce the number of candidate risk predictors. Nearly half of the studies in our review reduced the initial number candidate risk predictors prior to the multivariable modelling, yet over half of these failed provide sufficient detail on how this was carried out.

The most commonly used strategy to build a multivariable model is to use an automated selection approach (forward selection, backward elimination or stepwise) to derive the final risk prediction model (50% in our review). Automated selection methods are data-driven approaches based on statistical significance without reference to clinical relevance, and it has been shown that these methods frequently produce unstable models, have biased estimates of regression coefficients and yield poor predictions [82-84].

Arguably, regardless of how the multivariable model is developed, all that ultimately matters is to demonstrate that the model works. Thus, after a risk prediction model has been derived, it is essential that the performance of the model be evaluated. Broadly speaking, there are three types of performance data one can present, in order of increasing levels of evidence: (1) apparent validation on the same data used to derive the model; (2) internal validation using a split sample (if the cohort is large enough), cross-validation or, preferably, resampling (that is, bootstrapping); and (3) external validation using a completely different cohort of individuals from different centres or locations than those used to derive the model [85,86]. Investigators in over half of the studies in our review (54%) conducted an external validation on cohorts that were much larger than other reporting in other reviews [72,87].

Reporting performance data solely from an apparent validation analysis is to a large extent uninformative, unless the obvious optimism in evaluating the performance based on the same data used to derive the model is accounted for and this optimism quantified (using internal validation techniques such as resampling). Unless the cohort is particularly large (> 20,000), then using a split sample to derive and evaluate a model also has limited value, especially if the cohorts are randomly split, since the two cohorts are selected to be similar and thus produce overly optimistic performance data. In models in which a split sample has been used, a better approach is a nonrandom split (that is, certain centres or a temporal split) [85,86].

### What is already known on the topic

The findings of this review are consistent with those of other published reviews of prediction models in cancer [3,70,71], stroke [4,73,88], traumatic brain injury [68,72], liver transplantation [5] and dentistry [89]. We observed poor reporting in all aspects of developing the risk prediction models in terms of describing the data and providing sufficient detail in all steps taken in building the model.

### Limitations

Our systematic review was limited to English-language articles and did not consider grey literature; therefore, we may have missed some studies. However, we strongly suspect that including articles in our review would not have altered any of the findings.

## Conclusions

This systematic review of 39 published studies highlights numerous methodological deficiencies and a generally poor level of reporting in studies in which risk prediction models were developed for the detection of prevalent or incident type 2 diabetes. Reporting guidelines are available for therapeutic [90], diagnostic [91] and other study designs [14,92,93], and these have been shown to increase the reporting of key study information [94,95]. Such an initiative is long overdue for the reporting of risk prediction models. We note that in the field of veterinary oncology, recommended guidelines for the conduct and evaluation of prognostic studies have been developed to stem the tide of low-quality research. Until reporting guidelines suitable for deriving and evaluating risk prediction models are developed and adopted by journals and peer reviewers, the conduct, methodology and reporting of such models will remain disappointingly poor.

## Competing interests

The authors declare that they have no competing interests.

## Authors' contributions

GSC contributed to the study design, carried out the data extraction of all articles and items, compiled the results and drafted the manuscript. SM contributed to the study design, duplicate data extraction and drafting of the article. OO and LMY carried out duplicate data extraction and commented on the manuscript. All authors read and approved the final manuscript.

## Authors' information

All authors are medical statisticians.

## Appendix 1: Search strings

### PubMed search string

'diabetes'[ti] AND ('risk prediction model'[tiab] OR 'predictive model'[tiab] OR 'predictive equation'[tiab] OR 'prediction model'[tiab] OR 'risk calculator'[tiab] OR 'prediction rule'[tiab] OR 'risk model'[tiab] OR 'statistical model'[tiab] OR 'cox model'[tiab] OR 'multivariable'[tiab]) NOT (review[Publication Type] OR Bibliography[Publication Type] OR Editorial[Publication Type] OR Letter[Publication Type] OR Meta-analysis[Publication Type] OR News[Publication Type]).

### EMBASE search string

risk prediction model.ab. or risk prediction model.ti. or predictive model.ab. or predictive model.ti. or predictive equation.ab. or predictive equation.ti. or prediction model.ab. or prediction model.ti. or risk calculator.ab. or risk calculator.ti. or prediction rule.ab. or prediction rule.ti. or risk model.ab. or risk model.ti. or statistical model.ab. or statistical model.ti. or cox model.ab. or cox model.ti. or multivariable.ab. or multivariable.ti. and diabetes.ti not letter.pt not review.pt not editorial.pt not conference.pt not book.pt.

## Pre-publication history

The pre-publication history for this paper can be accessed here:

http://www.biomedcentral.com/1741-7015/9/103/prepub
